# *Streptococcus sputorum*, a Novel Member of *Streptococcus* with Multidrug Resistance, Exhibits Cytotoxicity

**DOI:** 10.3390/antibiotics10121532

**Published:** 2021-12-14

**Authors:** Chao Wang, Yuan Zeng, Mengyu Wei, Lanqing Cui, Yuqin Song, Gang Zhang, Yun Li, Jie Feng

**Affiliations:** 1 State Key Laboratory of Microbial Resources, Institute of Microbiology, Chinese Academy of Sciences, Beijing 110101, China; wangchao@im.ac.cn (C.W.); zengyuan19@mails.ucas.ac.cn (Y.Z.); vmyonly@163.com (M.W.); songyq@im.ac.cn (Y.S.); zhanggang@im.ac.cn (G.Z.); 2 College of Life Science, University of Chinese Academy of Sciences, Beijing 110101, China; 3 College of Life Science, Hebei University, Baoding 071000, China; 4 Institute of Clinical Pharmacology, Peking University First Hospital, Beijing 100083, China; cuilanqing@bjmu.edu.cn

**Keywords:** cytotoxicity, multidrug resistance, novel species of *Streptococcus*, whole-genome phylogenetic analysis

## Abstract

We describe the genomic and phenotypic characteristics of a novel member of *Streptococcus* with multidrug resistance (MDR) isolated from hospital samples. Strains SP218 and SP219 were identified as a novel *Streptococcus*, *S*. *sputorum*, using whole-genome sequencing and biochemical tests. Average nucleotide identity values of strains SP218 and SP219 with *S*. *pseudopneumoniae* IS7493 and *S. pneumoniae* ST556 were 94.3% and 93.3%, respectively. Genome-to-genome distance values of strains SP218 and SP219 with *S*. *pseudopneumoniae* IS7493 and *S. pneumoniae* ST556 were 56.70% (54–59.5%) and 56.40% (52.8–59.9%), respectively. The biochemical test results distinguished these strains from *S*. *pseudopneumoniae* and *S. pneumoniae*, particularly hydrolysis of equine urate and utilization of ribose to produce acid. These isolates were resistant to six major classes of antibiotics, which correlated with horizontal gene transfer and mutation. Notably, strain SP219 exhibited cytotoxicity against human lung epithelial cell line A549. Our results indicate the pathogenic potential of *S*. *sputorum*, and provide valuable insights into mitis group of streptococci.

## 1. Introduction

The mitis group of streptococci (MGS) consists of 20 currently described *Streptococcus* species that cause a wide range of infections in humans, including pneumonia, infective endocarditis, and septicemia [1,2,3]. Some MGS strains are highly virulent with various pathologies, such as *Streptococcus* *pneumoniae*, whereas others are low-pathogenicity commensal species.

Discrimination among MGS bacteria is important for obtaining accurate epidemiological information about MGS and their clinical significance, as their pathogenic potentials and sensitivities to various drugs differ considerably [4,5]. With the decreasing cost of genome sequencing, MGS can be classified more precisely through whole-genome sequencing [3,6]. Additionally, the identification of novel numbers, such as *Streptococcus pseudopneumoniae* [7], *Streptococcus tigurinus* [8], and *Streptococcus dentisani* [9], supplemented the understanding of MGS and avoided the misdiagnosis in clinical settings. In this study, we performed whole-genome phylogenetic analysis and identified a novel species of MGS which were collected from a hospital in Beijing in 2015. According to the phenotypic and phylogenetic data, SP218 and SP219 represent a novel species in the genus *Streptococcus*, for which the name *Streptococcus sputorum* sp. nov. is proposed. Remarkably, it was multidrug resistance and exhibited cytotoxicity against the human lung epithelial cell line A549. Our data demonstrated that the whole-genome analysis is an effective way to accurately identify the novel species in MGS, and the characterization of those novel species enabled a more in-depth acknowledgement of MGS diversity. Further analysis of the whole genome sequencing result found that this MDR species contains a lot antibiotic resistant genes and resistance mutations. It should be a part of the antibiotic resistance network of MGS.

## 2. Results and Discussion

### 2.1. Phylogenetic Analysis of the Isolates

**Figure 1:**
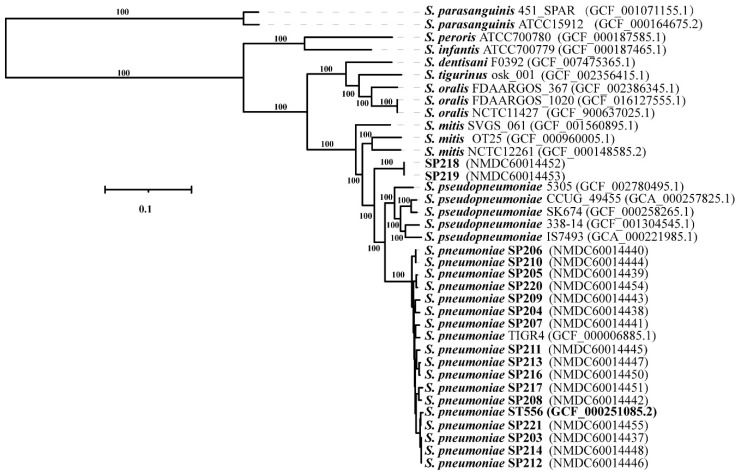
Whole-genome maximum-likelihood phylogenetic tree constructed using 687 core genome sequences from 18 clinical isolates and 19 genomes obtained from Refseq belonging to MGS. The sequences of *S*. *parasanguinis* ISU2812 and *S*. *parasanguinis* NCTC10234 were used as the root. Numbers at major branches represent bootstrap values based on 100 replications. The bar indicates changes per nucleotide position.

We collected 18 strains of *Streptococcus* sp. that were isolated from sputum samples of emergency ward patients in a hospital in Beijing in 2015 (7 females and 11 males). The 18 strains were initially identified as *S. pneumoniae* using the VITEK-2 system (BioMérieux, Marcy l’Etoile, France). To clarify their phylogenetic relationships, Illumina sequencing was performed and a maximum-likelihood phylogenetic tree of the 18 strains was constructed based on the concatenated sequences of 687 core genes, along with genome sequences for 19 representative species [3] of MGS downloaded from RefSeq (Figure 1). Sixteen strains clustered with *S. pneumoniae*, while the other two strains (SP218 and SP219), which were isolated from different patients, constituted a distinct lineage from *S. pneumoniae* and *S*. *pseudopneumoniae*. Average nucleotide identity (ANI) values between strain SP218 and SP219 and between *S*. *pseudopneumoniae* IS7493 and *S. pneumoniae* ST556 were 94.3% and 93.3%, respectively. Genome-to-genome distance (GGD) values between strain SP218 and SP219 and between *S*. *pseudopneumoniae* IS7493 and *S. pneumoniae* ST556 were 56.70% (54–59.5%) and 56.40% (52.8–59.9%), respectively. The threshold values of ANI and GGD for species discrimination are 95–96% and 70%, respectively. The SP218 and SP219 genomes contain 2,267,731 and 2,267,305 nucleotides, respectively, and both have GC contents of 39.74 mol%, within the range of values for *Streptococcus* species (33.8–43.4 mol%) [10]. The isolates SP218 and SP219 showed identical ANI and GGD values for species discrimination, confirming that these isolates represent the same species. These results suggest that strain SP218 and SP219 represent a novel species belonging to genus *Streptococcus*.

### 2.2. Morphological and Physiological Characterization of the New Species

Colonies of strains SP218 and SP219 on sheep blood agar were alpha-hemolytic, smooth, and white to grayish with a diameter of 0.5–1 mm after incubation at 37 °C with CO_2_ for 24 h (Figure S1A,D). The cells formed chains in broth culture (Figure S1B,E). As demonstrated using transmission electron microscopy, the strains do not have capsules (Figure S1C,F), as is the case for *S*. *pseudopneumoniae* [7]. However, in contrast to *S*. *pseudopneumoniae*, SP218 and SP219 were susceptible to optochin. The inhibition zones was 18 mm (>14 mm) when incubated under an atmosphere of elevated CO_2_ or ambient conditions. SP218 and SP219 can dissolve the bile, showed a OD-value 0.2, which differed from *S. pseudopneumoniae* (showed a median OD-value 1.8 [7]).

To classify the new species through physiological characterization, we tested the strains using the Rapid ID 32 Strep system, which can be used to differentiate among most *Streptococcus* species for which phenotypic characteristics have been described. The biochemical characteristics of the two isolates were represented by Rapid ID 32 Strep profiles of 4–2–1–7–2–4–1–1–1–0–0 and 0–2–1–0–2–4–1–1–1–0–0, respectively. These results do not align with any species that the Rapid ID 32 Strep system can classify. Some traits clearly distinguished these strains from their nearest phylogenetic relatives (*S*. *pseudopneumoniae* and *S. pneumoniae*), as shown in Table S1. Strains SP218 and SP219 hydrolyzed equine urate and utilized ribose to produce acid, whereas *S*. *pseudopneumoniae* and *S. pneumoniae* did not. SP219 exhibited β-galactosidase activity (Table S1). Given the phenotypic and phylogenetic data described above, we propose that SP218 and SP219 belong to a novel species of *Streptococcus*: *Streptococcus sputorum* sp. nov. 

### 2.3. Antibiotic Resistance via HGT

Antibiotic susceptibility profiles were determined for all 18 clinical isolates (Table 1). Strains SP218 and SP219 were MDR, as they were not susceptible to six major classes of antibiotics: β-lactams, erythromycin, fluoroquinolone, tetracycline, trimethoprim, and sulfamethoxazole. They were, however, susceptible to clindamycin. The strains showed broader resistance than 10 of the tested *S. pneumoniae* isolates, and had higher resistance level to fluoroquinolone than other *S. pneumoniae* isolates in this study.

**Figure 2:**
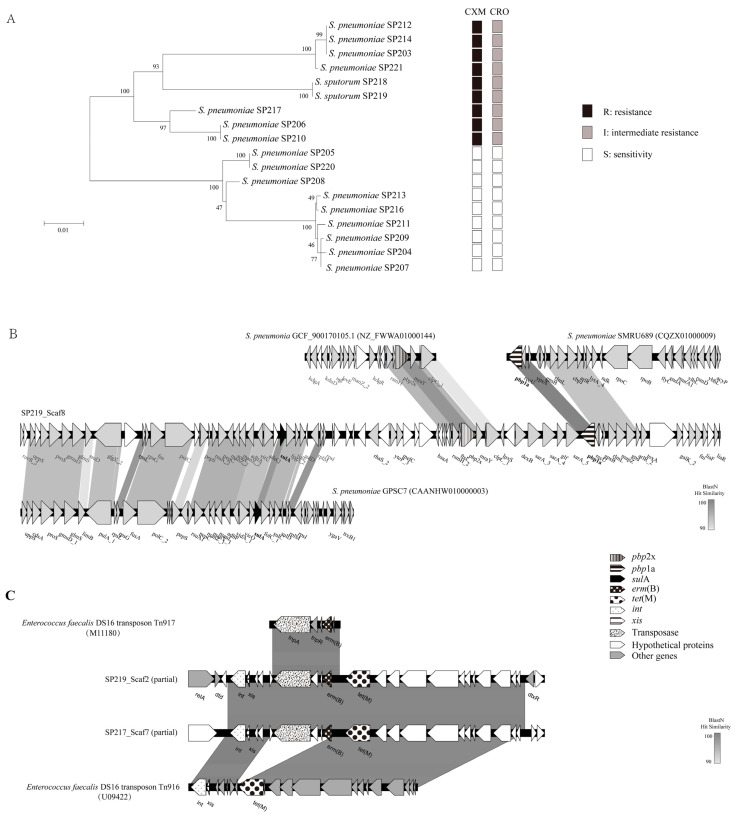
(**A**) Maximum-likelihood tree generated using the MEGA software based on the serial amino acid sequences of PBP1a and PBP2x from tested clinical isolates, and their cephalosporin resistance profiles. Bootstrap values of 1000 replications are shown. The bar indicates changes per amino acid position. Black squares: resistance; gray squares: intermediate resistance; white squares: sensitivity. (**B**) Genomic organization of *pbp*1a-, *pbp*2x-, and *sul*A-carrying scaffolds showing highest homology with *S. pneumoniae* and comparison with related sequences available in GenBank. Genes are labeled and are textured based on functional classification, as shown in the key. Gray shading represents similarity at the nucleotide level. (**C**) Genomic organization of Tn*3872* of strain S219 and *S. pneumoniae* SP217, and comparison with related sequences available in GenBank. Genes are labeled and are textured based on functional classification, as shown in the key. Gray shading represents similarity at the nucleotide level.

To clarify the mechanisms through which this new species acquired resistance, we analyzed genes associated with antibiotic resistance. The *S. pneumoniae* genome encodes six penicillin-binding proteins (PBPs), among which PBP2x and PBP1a contribute to cephalosporin resistance [11]. A maximum-likelihood tree was constructed from serial amino acid sequences of PBP1a and PBP2x from tested clinical isolates to identify variation in these proteins. As shown in Figure 2A, the amino acid sequences of PBP1a and PBP2x from the 18 clinical isolates in this study clustered into two highly supported monophyletic clades (with 100% bootstrap confidence). All isolates in the clade containing SP218 and SP219 were insensitive to cephalosporins (cefuroxime and ceftriaxone), while the other clade exhibited cephalosporin sensitivity. SP218 and SP219 clustered with non-susceptible β-lactam isolates; this clustering did not match the phylogenetic relationships observed for the core genome, indicating the occurrence of interspecies recombination events. Further evidence of gene transfer comes from GenBank, as the *pbp*1a sequence obtained here was identical to that of *S. pneumoniae* SMRU689 (CQZX01000009) (Figure 2B). Moreover, *pbp*2x was highly homologous (99.07%) to the corresponding gene of *S. pneumoniae* GCF_900170105.1 (NZ_FWWA01000144). A fragment with greater than 90% nucleotide similarity to *S. pneumoniae* GPSC7 (CAANHW010000003) was found in the region approximately 20 kb upstream of *pbp*2x, which contained a *sul*A gene with mutations conferring resistance to sulfonamides. These data strongly suggest that the new species, *S. sputorum*, obtained gene fragments from *S. pneumoniae* and recombined them into its genome. It consistent with previous reports, gene exchanges were occur frequently among this group [12]. Recombination among *S. pneumoniae*, *Streptococcus mitis*, and *Streptococcus oralis* contribute to the development and dissemination of resistance to β-lactam antibiotics [13,14].

SP218 and SP219 acquired erythromycin and tetracycline resistance through integration of Tn*3872* belonging to the Tn*916* family [15], which is widely distributed in *S. pneumoniae*. Tn*916* family genes were found in all 18 isolates analyzed in this study. SP218 and SP219 contained Tn*3872* sequences identical to that of the isolate *S. pneumoniae* SP217 (Figure 2C). These results suggest that horizontal gene exchange has been involved in the acquisition of resistance by these clones. Thus, this study provides evidence of interspecies genetic transfer between the new species and *S. pneumoniae*.

All *S. pneumoniae* in this study were sensitive to fluoroquinolone except SP217. Relative to *S. pneumoniae* SP217, the strains SP218 and SP219 displayed higher resistance level to fluoroquinolone. Mutations in GyrA (S81Y and S114G) and ParC (S52G, D56E, S79I, and N91D) confer resistance to fluoroquinolone in *S. pneumoniae* [16]. These mutations were also found in GyrA and ParC of SP218 and SP219. Furthermore, GyrA and ParC of SP218 and SP219 shared 97.89% and 97.04% idenity with the proteins in *S. streptococcus* SP217 and there could be unknown mutation involved in fluoroquinolone resistance in strains of the novel species.

### 2.4. Virulence Genes and Cytotoxicity of the New Species

To gain insights into the genetic features that promote adhesion, virulence, and colonization in these strains, we searched the Virulence Factor Database [17] for orthologs of virulence genes. SP218 and SP219 harbored 21 virulence genes in common with the reference *S. pneumoniae* genome (Table S2), including genes associated with adherence, enzymes, iron uptake, manganese uptake, and pneumolysin toxins, which have hemolytic activity. These findings indicate the pathogenic potential of these microorganisms. However, strains SP218 and SP219 did not harbor the capsule gene cluster, suggesting that they do not generate capsules. This is consistent with our transmission electron microscopy results, described above (Figure S1).

**Figure 3:**
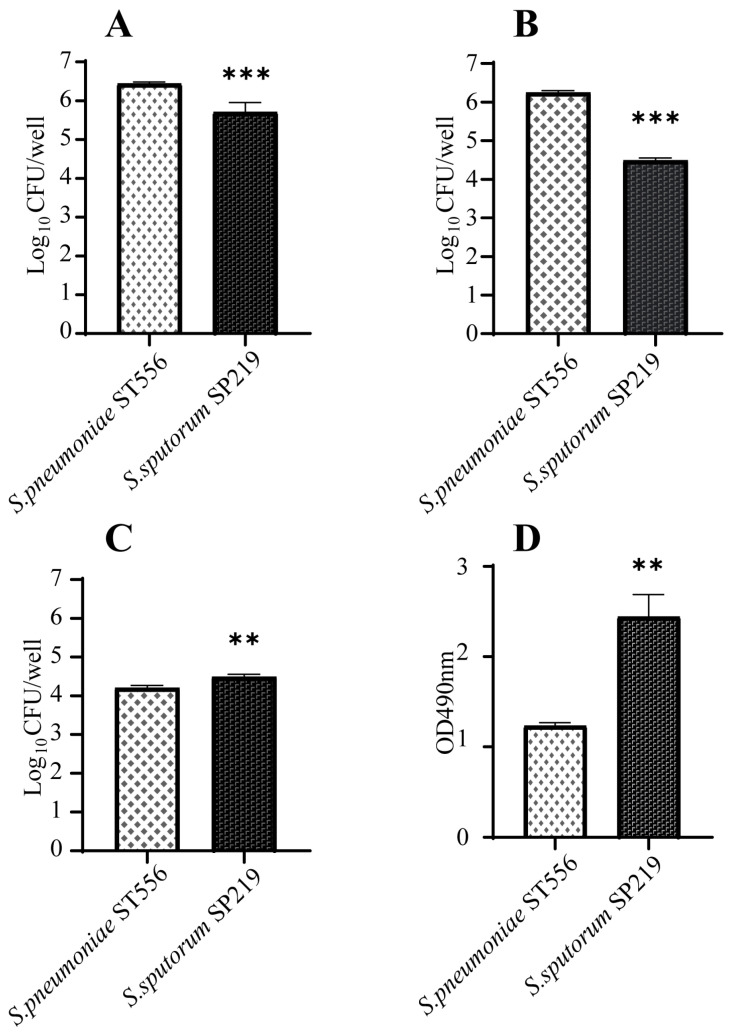
Interactions between A549 cells and *S*. *sputorum* strains. The bacterial suspensions were inoculated onto washed A549 cell monolayers and incubated for 2 h at 37 °C under 5% CO_2_ for adhesion assays (**A**). Bacterial invasion was also monitored after incubation for 2 h and no bacterial invasion was observed. The bacterial suspensions were inoculated onto washed A549 cell monolayers and incubated for 4 h at 37 °C under 5% CO_2_ for invasion (**B**) and cytotoxicity assays (**C**,**D**). For the cytotoxicity assays, (**C**) number of adherable living A549 cells after invasion assays, (**D**) LDH release test. The multiplicity of infection was approximately 50:1. *p*-values <0.001 (***), *p*-values <0.01 (**).

Interactions between pathogens and host mucosal epithelial cells are prerequisites for pneumococcal disease development. Therefore, we investigated the ability of strain SP219 to adhere to and invade A549 cells. As shown in Figure 3, it adhered to and invaded A549 cells. However, the efficiencies of adhesion and invasion were significantly lower for un-encapsulated strain SP219 than encapsulated *S. pneumoniae* ST556. We also examined the cytotoxicity of SP219 to A549 cells based on lactate dehydrogenase (LDH) release. SP219 infection caused significantly higher levels of cell necrosis and LDH release than *S. pneumoniae* ST556 infection in A549 cells. This suggests that SP219 could more effectively breach the first line of host defenses and damage human lung epithelial cells.

## 3. Conclusions

*S. pneumoniae* is a clinically important pathogen of the respiratory tract that causes various invasive infections. The closely related species *S*. *pseudopneumoniae* was first described in 2004 [7], and an increasing number of reports indicate that *S*. *pseudopneumoniae* is an emerging pathogen [2,18,19]. Therefore, the need to distinguish among *Streptococcus* species in clinical isolates is growing. Whole-genome analysis is a useful strategy for improving the accuracy of *Streptococcus* identification and classification. Our phenotypic and phylogenetic data clearly differentiate the two novel strains from *S. pneumoniae*. The presence of virulence genes and cytotoxicity factors suggests that *S*. *sputorum* could be pathogenic. Multiple HGT events resulted in the MDR phenotype of this new species. SP218 and SP219 were isolated from different patients at the same hospital, indicating the potential infectivity and transmissibility of *S*. *sputorum*. Thus, our study provides important insights into MGS.

## 4. Materials and Methods

### 4.1. Bacterial Strains, MIC Determination, and Morphological and Physiological Characterization of the Novel Strain

The 18 clinical strains were isolated for sputum samples by performing dilution plates. The purified strains were stored at −80 °C, and grown in brain heart infusion broth (BHI, BD, USA) or modified trypticase soy agar (TSA, BD, USA) containing 5% defibrinated sheep blood under a 5% CO_2_ atmosphere at 37 °C for 18–24 h prior to use. The 18 strains were initially identified as *S. pneumoniae* using the VITEK-2 system (BioMérieux, Marcy l’Etoile, France). The minimum inhibitory concentrations (MICs) of antibiotics were determined using the broth microdilution method according to Clinical and Laboratory Standards Institute guidelines (http://www.clsi.org/; accessed on 10 May 2021). A JEM-1400 transmission electron microscope (Jeol, Minato-Ku, Japan) and an SU1080 scanning electron microscope (Hitachi, Tokyo, Japan) were used to observe cell morphology, size and capsule growth state. For bile solubility testing, an inoculum was prepared from colonies taken from blood agar plates incubated overnight (37 °C) using a tube containing 2 mL saline with cell density adjusted to that of the McFarland 4 standard (approximately 1.5 × 10^9^ cells/mL). Then the suspension was divided equally into two tubes, with each containing each 1 mL. Then, 200 µL 10% sodium deoxycholate was added to the test tube and 200 µL saline was added to the control tube. Both tubes were incubated for 10 min at 37 °C, and then their absorbances were measured. The difference in absorbance between the test tube and control tube was calculated as an OD value. A negative difference between the test tube and the blank control was set to 0.0 OD value [20]. API Rapid ID 32 strips (BioMerieux, Marcy I’Etiole, France) were used for biochemical testing of the novel and reference strains, following the manufacturer’s instructions.

### 4.2. Whole-Genome Sequencing and Genome Analysis

Total DNA was purified using the TIANamp Genomic DNA Kit (Tiangen, Beijing, China) following the manufacturer’s instructions. Genome sequencing of all isolates in this study was performed using the Illumina HiSeq 2000 system (Illumina, San Diego, CA, USA). Coverage was 200-fold and the scaffold N50 value was approximately 200 kb. Assembly was performed using Spades software [21]. Predicted protein-coding sequences were detected using the NCBI Prokaryotic Genomes Automatic Annotation Pipeline (prokka 1.14.6). Antibiotic resistance genes were named in accordance with The Comprehensive Antibiotic Resistance Database and confirmed manually through Basic Local Alignment Search Tool (BLAST) and literature searches. Virulence genes were identified in accordance with the Virulence Factor Database [22] for orthologs of virulence genes. To clarify their phylogenetic relationships, a maximum-likelihood (ML) phylogenetic tree was constructed using RAxML version 8.2.12 [23]. The ML tree was constructed based on the concatenated sequences of 211 core genes, along with reference *Streptococcus* genome sequences downloaded from GenBank. Linear genetic diagrams were constructed using Easyfig version 2.1 [24]. Sequence comparisons were generated using mafft v7.487. Pairwise ANI values were estimated by calculating the average identity value of all BLAST results between each pair of genomes [25]. GGD was assessed using the web service of the Genome-to-genome Distance Calculator 2.1 [26]. Phylogenetic trees of the serial sequences PBP1a and PBP2x were constructed and genetic distances were determined using MEGA X [27].

### 4.3. Cell Adhesion and Invasion Assays

Cell adhesion and invasion assays had been modified from previously described [28]. For the cell adhesion assay, the strains were inoculated into BHI culture medium and grown at 37 °C to the middle logarithmic phase (optical density at 600 nm, OD_600_ = 0.5). Then, 5 × 10^6^ colony-forming units (CFUs) bacteria were added to 1 × 10^5^ cultured cells in each well (multiplicity of infection, approximately 50:1). Bacteria and cells were incubated for 120 min at 37 °C. At the end of incubation, the cells were extensively rinsed with phosphate-buffered saline five times and then incubated with 0.25% trypsin and 400 μL 0.025% Triton-X100 for 5 min. The cells were removed from the well. The suspension was serially diluted and plated onto TSA plates containing 5% sheep blood, which were incubated at 37 °C under 5% CO_2_ for 17–18 h for CFU determination. Adherent cells were counted at the end of each experiment. For each experiment, six replicates were assessed at least three times. Representative experiments are presented in the figures. For the cell invasion assay, all of the initial steps were the same as those used in the adhesion assay, except that bacteria and cells were incubated together for 4 h and penicillin (100 μg/mL) and gentamicin (50 μg/mL) were added to the culture medium for 60 min at the end of incubation to kill the bacteria outside of the cell. The MIC of SP218 and 219 to penicillin and gentamycin were 1 μg/mL.

### 4.4. LDH Release Test

The fresh strains were inoculated into BHI culture medium and cultured at 37 °C to the middle logarithmic phase (OD_600_ = 0.5). Then, 5 × 10^6^ CFUs bacteria were added to 1 × 10^5^ cultured cells in each well (multiplicity of infection, approximately 50:1). Bacteria and cells were incubated for 4 h at 37 °C. After incubation, the supernatant was centrifuged at 400× *g* for 5 min. The LDH Cytotoxicity Assay Kit (Beyotime, Shenzhen, China) was used for cytotoxicity testing of the novel and reference strains, following the manufacturer’s instructions. Then the absorbance was measured at 490 nm. Dual-wavelength determination was performed using any wavelength of 600 nm or greater as the reference wavelength.

## Figures and Tables

**Table 1:** Antibiotic susceptibility profiles of the clinical isolates in this study *.

National Microbiology Data Center Accession Number	Strain Number	Serotype	AMX	CXM	CRO	CPM	ERY	AZM	CLI	CIP	LVX	MXF	TC	TMP	SMZ
NMDC60014437	SP203	271	2	4	1	1	256	256	512	1	1	0.125	16	16	128
NMDC60014438	SP204	unannotated	0.031	0.008	0.008	0.031	256	256	512	0.25	0.5	0.125	64	1	512
NMDC60014439	SP205	unannotated	0.008	0.016	0.031	0.062	256	256	512	1	1	0.125	64	128	1024
NMDC60014440	SP206	81	1	4	0.5	1	256	256	512	1	1	0.125	64	64	512
NMDC60014441	SP207	505	0.016	0.016	0.008	0.031	256	256	512	1	1	0.125	32	2	32
NMDC60014442	SP208	342	0.031	0.062	0.031	0.062	256	128	256	0.25	0.5	0.062	32	16	32
NMDC60014443	SP209	unannotated	0.016	0.016	0.008	0.031	256	256	512	1	1	0.125	64	128	128
NMDC60014444	SP210	81	1	4	0.5	1	256	256	512	1	1	0.125	32	64	128
NMDC60014445	SP211	unannotated	0.031	0.016	0.016	0.031	128	256	512	1	1	0.125	32	64	128
NMDC60014446	SP212	271	1	4	0.5	1	256	256	512	0.25	0.5	0.125	16	64	>1024
NMDC60014447	SP213	7751	0.008	0.016	0.008	0.062	256	256	512	1	1	0.25	16	2	32
NMDC60014448	SP214	271	1	4	1	1	256	256	512	2	1	0.25	16	32	128
NMDC60014450	SP216	6946	0.016	0.031	0.016	0.062	128	256	512	1	1	0.125	16	1	128
NMDC60014451	SP217	3173	1	4	0.5	1	128	256	512	8	8	2	32	8	>1024
NMDC60014454	SP220	10085	0.031	0.25	0.031	0.031	256	256	512	0.5	0.5	0.125	16	16	32
NMDC60014455	SP221	9104	0.5	2	0.5	1	16	2	0.125	0.25	0.5	0.125	32	1	512
NMDC60014452	SP218	unannotated	1	2	0.25	1	64	128	0.25	64	8	4	32	64	>1024
NMDC60014453	SP219	unannotated	1	2	0.25	1	64	128	0.25	64	8	4	32	64	>1024
Total number of insensitive isolates			0	9	9	0	18	18	15	4	3	3	18	18	18
Rate of insensitive isolates			0	50%	50%	0	100%	100%	83.33%	22.22%	16.67%	16.67%	100%	100%	100%

AMX, Amoxicillin; CXM, cefuroxime; CRO, ceftriaxone; CPM, cefepime; ERY, erythromycin; AZM, azithromycin; CLI, clindamycin; CIP, ciprofloxacin; LVX, levofloxacin; MXF, moxifloxacin; TC, tetracycline; TMP, trimethoprim; SMZ, sulfamethoxazole. * The MICs (mg/L) for strains were determined using broth microdilution assays according to CLSI (Clinical and Laboratory Standards Institute [http://www.clsi.org/; accessed on 10 May 2021]).

## Data Availability

All datasets were provided by requests to the author for correspondence.

## Supplementary Materials

The following are available online at https://www.mdpi.com/article/10.3390/antibiotics10121532/s1, Table S1: Characteristics used to distinguish strains SP218 and SP219 from *S. pneumoniae* and *S. pseudopneumoniae*. Table S2: Virulence factor genes in the genomes of SP218, SP219, and the reference genome of *S. pneumoniae* annotated using the Virulence Factor Database (http://www.mgc.ac.cn/VFs/; accessed on 19 March 2021). Figure S1: (A,D) Images of the colony morphology of SP218 and SP219. Bar = 2 mm; (B,E) microscopy images of SP218 and SP219. Bar = 10 µm; (C,F) transmission-electron-microscope images of SP218 and SP219. Bar = 1 µm. SP218 and SP219 grown at 37 °C on TSA medium containing 5% defibrinated sheep blood in a 5% CO_2_ atmosphere. Reference [29] is cited in the supplementary materials.

## Nucleotide Sequence Accession Numbers

The draft genome sequence of isolates have been deposited in National Microbiology Data Center under the accession number NMDC60014437 to NMDC60014448, NMDC60014450 to NMDC60014455.
