# Supplementary material for: Streptococcus sputorum, a Novel Member of Streptococcus with Multidrug Resistance, Exhibits Cytotoxicity
Source: Antibiotics (Basel). 2021 Dec 14;10(12):1532. doi: 10.3390/antibiotics10121532 (PMC8698525; doi:10.3390/antibiotics10121532)
Supplement: Supplementary file 1 [file antibiotics-10-01532-s001.zip › antibiotics-1464515-supplementary/Table S1.pdf]

Table S1 Characteristics used to distinguish strain SP218 and SP219 from *S.pneumoniae* and *S.pseudopneumoniae*

Characteristic	SP218	SP219	S. *pneumoniae* ST556	*S. pseudopneumoniae**
Temperature (optimum)℃	37	37	37	37
pH (optimum)	7.2	7.2	7.2	7.2
Sodium deoxycholate	+	+	+	-
Optochin	+	+	+	+
Enzyme activity				
Arginine dihydrolase	-	-	-	-
β-glucosidase	-	-	-	-
β-galactosidase	-	+	-	-
β-glucuronidase	-	-	-	-
α-galactosidase	+	+	+	+
Alkaline phosphatase	-	-	-	-
Alanyl-phenylalanyl-proline	+	+	+	-
Arylamidase				
β-galactosidase	-	-	-	-
Pyroglutamic acid arylamidase	-	-	-	-
n-acetyl- *β*-glucosaminidase	-	-	-	-
Glycyl-tryptophan arylamidase	+	+	+	-
β-mannosidase	-	-	-	-
Acid production				
Ribose	+	+	-	-
Mannitol	-	-	-	W
Sorbitol	-	-	-	-
Lactose	W	+	W	+
Trehalose	W	+	+	-
Raffinose	-	+	+	-
Glycogen	-	-	-	-
Pullulane	-	-	-	-
Maltose	+	+	+	-
Melibiose	-	-	-	-
Saccharose	-	-	-	-
L-arabinose	+	+	+	-
D-arabitol	-	-	-	-
Melezitose	-	-	-	-
methyl-*β* D-glucopyranoside	-	-	-	-
Tagatose	-	-	-	-
Cyclode Xtrin	-	-	-	-
Else				
Acetoin production	-	-	-	-
Hydrolysis of hippurate	+	+	-	-

*Lim YK, Park SN, Shin JH, Chang YH, Shin Y, Paek J, Kim H, Kook JK. 2019. *Streptococcus chosunense* sp. nov., Isolated from Human Postoperative Maxillary Cyst. Curr Microbiol 76:1193-1198. https://doi.org/10.1007/s00284-019-01746-0

Symbols:+, positive;−, negative; w,weak.
