# Supplementary figures and images for: Streptococcus sputorum, a Novel Member of Streptococcus with Multidrug Resistance, Exhibits Cytotoxicity
Source: Antibiotics (Basel). 2021 Dec 14;10(12):1532. doi: 10.3390/antibiotics10121532 (PMC8698525; doi:10.3390/antibiotics10121532)

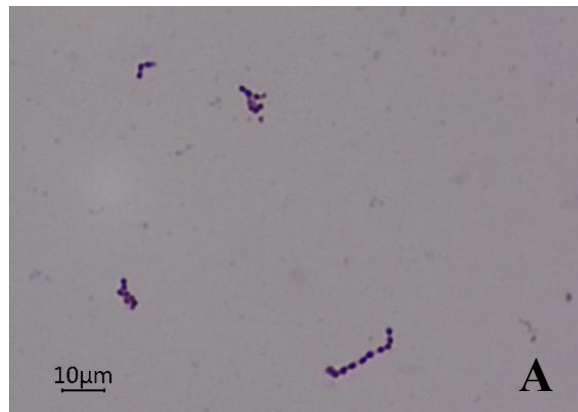

**ST556**

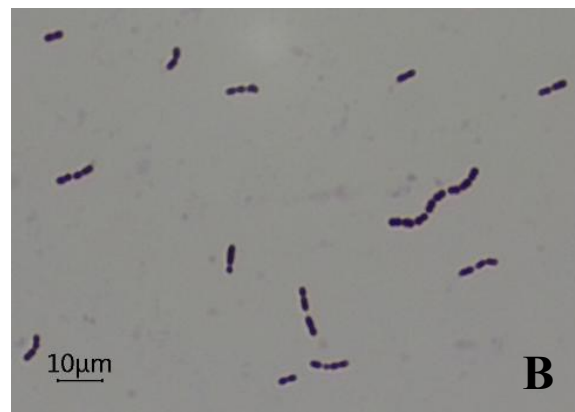

**SP218**

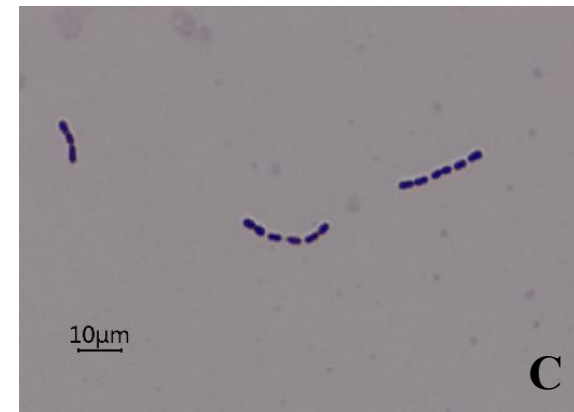

**SP219**

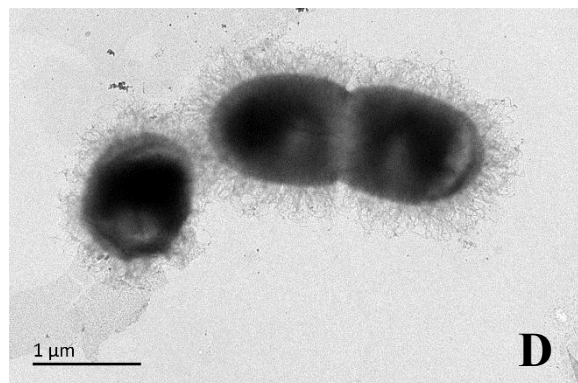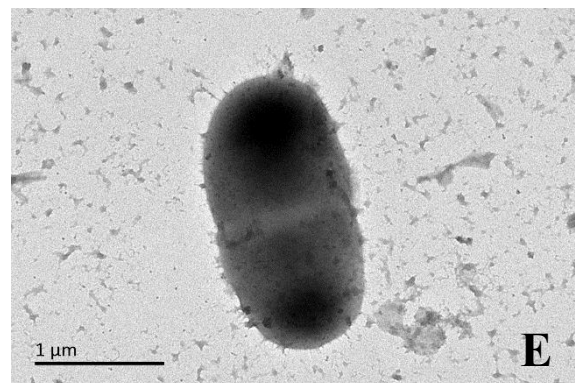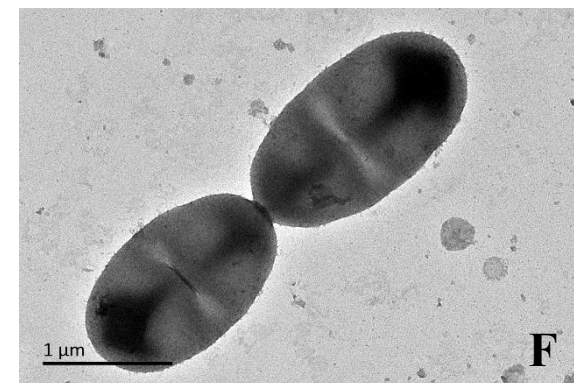

Supplement: Supplementary file 1 [file antibiotics-10-01532-s001.zip › antibiotics-1464515-supplementary/Figure S1.pdf]
